# Supplementary material for: A qPCR assay for the detection of Phytophthora abietivora, an emerging pathogen on fir species cultivated as Christmas trees
Source: PLoS One. 2025 Apr 2;20(4):e0320680. doi: 10.1371/journal.pone.0320680 (PMC11964239; doi:10.1371/journal.pone.0320680)
Supplement: Attachment — Submitted filename: Response_to_Reviewers_Charron_et_al_2024.docx [file pone.0320680.s009.docx]

**Response to Reviewers**

**Journal requirements:**

**When submitting your revision, we need you to address these additional requirements.**

**Please ensure that your manuscript meets PLOS ONE's style requirements, including those for file naming.**

Answer: Style requirements have been respected.

**Role of funders in financial disclosure:**

Answer: We added the financial disclosure section to the manuscript

**We note that Figure 2 in your submission contain [map/satellite] images which may be copyrighted. We require you to present written permission from the copyright holder to publish these figures specifically under the CC BY 4.0 license.**

Answer: The data used to create the maps in Figure 2 with R programing are already under CC BY 4.0 copyright license and can be used as is with the proper citation. Here is the full reference :

MINISTÈRE DES RESSOURCES NATURELLES ET DES FORÊTS. Base de données géographiques et administratives, [Jeu de données], dans Données Québec, 2012, mis à jour le 10 septembre 2024. [Https://www.donneesquebec.ca/recherche/dataset/base-de-donnees-geographiques-et-administratives], (consulté le 20 novembre 2024).

the following text has been included and the citation #37 also refers to this source: “The data used to create these maps was obtained from the Ministry of Natural Resources and Forests under a CC BY 4.0 license, with permission from MNRF, original copyright 2024. [https://www.donneesquebec.ca/recherche/dataset/base-de-donnees-geographiques-et-administratives]”

**Please review your reference list to ensure that it is complete and correct.**

Answer: References have been reviewed.

**Reviewer #1:**

**This manuscript is about the development of a qPCR to detect *Phytophthora abietivora* which is an emerging pathogen of Christmas tree (firs) in North America. The authors discuss the development of the assay in sufficient detail and discuss the results in the context of its future application as a monitoring and detection tool. The quality of the work in this manuscript appears sound. The qPCR assay is specific to the *P. europaea* complex so the authors need to be careful to state it being species specific when they themselves declare it semi specific. I would recommend this manuscript be accepted with minor changes.**

Answer: We thank the reviewer for his constructive comments and general appreciation of our manuscript. We did our best to clearly state the limitations of our assay in order to avoid misleading the readership into thinking that our assay is specific

**Line 21 - Provide example of Phytophthora disease of a natural ecosystem which threatens biodiversity, or write something like “global plant biodiversity, horticulture and trade.”**

Answer: we changed the sentence as suggested.

Line 20-21 […] “Emerging species of the Phytophthora genus are among the most important threats to global plant biodiversity, horticulture and trade.” […]

**Line 21 – species name for Christmas trees**

Answer: We added the species names (both common and latin) to the sentence.

Line 21-23 […] “For instance, Phytophthora root rot (PRR) of Christmas trees, mainly Fraser (Abies fraseri) and balsam firs (Abies balsamea), is responsible for 10% of the observed mortality in nurseries.” […]

**Line 26 – remove the word generation**

Answer: The word was removed.

Line 26-27 […] “For these applications, molecular detection of environmental DNA (eDNA) provides the fast and high-throughput results needed.” […]

**Line 27 – scientific name for firs**

Answer: We added the scientific name for the fir genus.

Line 28 […] “*Phytophthora abietivora* was associated with PRR in firs (*Abies spp.)* cultivated as Christmas trees in the province of Québec (Canada).” […]

**Line 32 – which species of firs were included?**

Answer: We added the names of the species included in the dataset.

Line 33-34 […] “The assay was tested on DNA obtained from healthy-looking and PRR symptomatic Fraser and balsam firs.” […]

**Line 37 – how many of those eDNA samples were from symptomatic trees and did the results correlate with baiting?**

Answer: 42 out of 68 (about 61%) were from symptomatic trees, only 3 out of 7 samples with positive *P. abietivora* baiting showed positive detection. We modified the abstract to incorporate the information.

The following sentence was removed:

While factors such as the tree species, the sampled region or the year of sampling did not affect the proportion of positive results, samples from trees showing PRR-like symptoms had significantly higher odds of testing positive compared to healthy-looking trees.

The original sentence was modified to

Line 37-40 […] Out of 488 DNA samples from soil, 68 were positive for *P. abietivora* from which 42 (61.7%) were from PRR symptomatic trees. Only a slight overlap (3 out of 7 samples) was observed with previously obtained baiting results. […]

**If it cross reacts with P. europaea, would you not report the result as positive for P. abietivora/P. europaea?**

Answer: We agree with the reviewer that other species from the *P. europaea* complex are expected to cross react with our assay. This limitation is clearly stated in the discussion (lines 425 to 438). In addition, the context of our study should allow us to exclude the presence of the other species thus allowing us to only refer to *P. abietivora*.

We modified the following sentence to provide more clarity about the use of only *P. abietivora* in the abstract:

The assay was shown to be semi-specific because it cross-reacted with *P. europaea*, and three other phylogenetically close species unrelated to fir diseases.

Was modified to:

Line 34-36 […] The assay was shown to be semi-specific because it cross-reacted with *P. europaea* and three other phylogenetically close species, but deemed specific in the context of PRR of firs. […]

**Line 47 – Reference 7 (O’Brien et al 2009) seems out of place here.**

Answer: this citation was removed from this sentence.

**Line 49 – include** **(genus Abies) after true firs.**

Answer: The sentence was modified as suggested.

Line 47-51 […] “For instance, one of the industries faced with Phytophthora root rot (PRR) is the cultivation of Christmas trees as ornamental plants, where PRR is one of the most prevalent and widely distributed diseases of true firs (genus *Abies*), killing about 10% of the planted Christmas trees in the United States of America [7].” […]

**Line 54 – roots not root**

Answer: Correction made

Line 56-59 […] “Current diagnosis of PRR in firs requires laborious isolation and identification of the causal agent, which is further challenged given the secondary colonization of infected roots and the outcompeting growth of other fungi and oomycetes on semi-selective isolation media [12].” […]

**Line 66 – species name for Fraser fir**

Answer: The species names for Fraser fir was added in a prior sentence along with the name for balsam fir as they are the first instance of the names in the introduction.

Line 65-68 […] “While *P. cinnamomi* is the main species causing PRR in the eastern United States [18], a recent survey identified *P. abietivora* as the species most frequently associated with dying balsam (*Abies balsamea*) and Fraser firs (*Abies fraseri*) cultivated as Christmas trees in Québec, Canada [19].” […]

**Line 83 – species instead of agents**

Answer: The sentence was modified according to the comment.

Line 86-88 […] “As such, many studies have developed specific qPCR assays targeting Phytophthora species, such as *P. cryptogea* [24], *P. ramorum* [13], *P. cactorum* [25] and many others [26,27].” […]

**Line 191 – company details for hemocytometer?**

Answer: Added details to the instrument description.

Line 198-200 […] Oospore count was estimated using an Improved Neubauer hemacytometer (American Optical Company, Buffalo, USA). […]

**Line 197 – garden soil collected from underneath what plants at what site? How did you know it was free of P. abietivora? Would there be other phytophthora present?**

We did additionnal lab work to validate the absence of *P. abietivora* in the garden soil. We performed the DNA extractions on 6 independent samples of the same soil sample used in the study and ran the assay in 3 replicates. None of the samples produced a positive result, confirming the absence of *P. abietivora* or the other genetically close species in the soil. We added two sentences about this control in the manuscript to clarify this. We can however not exclude the presence of other *Phytophthora* species.

Line 212-215 […] “To make sure this soil was not previously contaminated with P. abietivora, six independent samples were used for DNA extractions with the Powersoil protocol. On each of the DNA samples obtained, the detection assay was performed in three technical replicates.”

Line 326-329: […] A control experiment conducted on the garden soil used for LOQ determination revealed that neither P. abietivora, nor any species from the P. europaea species complex, were present before the addition of the oospores, as all six soil samples returned negative detection results. […]

**Line 199 – did you use the Powersoil pro kit for the extractions with soil?**

Answer: Yes, we used the DNeasy® Powersoil pro kit (QIAGEN, Hilden, Germany). Text has been clarified.

Line 207-209 […] “For DNA extractions of oospores mixed with soil, the oospores were added to a PowerBead pro tube containing 250 mg of garden soil free from *P. abietivora*, and DNA was extracted following the DNeasy® Powersoil pro kit protocol (QIAGEN, Hilden, Germany).” […]

**Line 243 – so the qPCR (primers and probe) target P. abievitora/P. europaea/P. flexuosa/P.tyrrenhica/P. uliginosa? If both P. abievirora and P. europaea are associate with Christmas trees I think this limitation should be discussed**

Answer: The reviewer is right, but there is already a part of the discussion dedicated to clarifying the situation:

Line 430-448 […] “The ITS as a marker provides resolution between distant species, but its use is sometimes challenged when dealing with closely related species [50]. For the P_abi_detect assay, this translates as being only semi-specific as the ITS2 sequence targeted by the primers and probe is identical between the five members of the *P. europaea* species complex, which includes *P. abietivora*, *P. flexuosa*, *P. uliginosa* and *P. tyrrhenica* [21]. This is not uncommon as other assays targeting *Phytopthora* pathogens have also showed cross-reactivity with other closely related species (P. cactorum [25], P. cinnamomi [51], P. infestans [45,52]). In those cases, the validity of the assay is preserved as species cross-reacting with the assay are often not found in the same environments as the targeted pathogen. For the P_abi detect assay this shortcoming is mitigated by the fact that, besides *P. europaea*, none of the other members of this complex has ever been reported in association with evergreen trees or reported in North America (*P. flexuosa*) [53], (*P. uliginosa*) [54], (*P. tyrrhenica*) [55]. Previous reports of *P. europaea* on North American firs are questionable since there are chances that they resulted from misidentification due to the high identity between the ITS sequences of P. europaea and *P. abietivora*. In cases for which isolates are available, sequencing of loci offering more discrimination will be needed for confirmation. The P_abi_detect assay has been developed to provide a tool for detection and diagnosis in North American Christmas tree productions. Remaining within this context excludes the presence of the other species from the P. europaea species complex. When the presence of other species from this complex cannot be excluded, this assay should always be coupled with confirmation methods such as strain baiting or strain isolation from diseased tissues, followed by Sanger sequencing of a discriminating locus to confirm the presence of only *P. abietivora*.” […]

Text within lines 438-440 were added to specify that cross reactions are not uncommon for other such assays.

**Line 260 – P.** **cinnamomic misspelled**

Answer: Correction made

Line 274-277 […] “No nonspecific amplification under the LOD95% cut-off was observed in the tested conditions for the genomic DNA of the 22 other species tested, including members of subclade 7a (*P. fragariae, P. ×cambivora,* and *P. cinnamomi*).” […]

**Line 261 – amplified misspelled**

Answer: Correction made

Line 278-280 […] “Following the criteria for positivity established above, three species (*P. cactorum, P. sansomeana* and the unknown *Phytophthora*) amplified at a late cycle, past the cut-off and for only for one replicate out of three, were not considered as positive and may be the result of a cross-contamination.” […]

**Line 301 – arguably false positives can be more detrimental as it reduces the reliability and confidence in the testing method, unless positive detections are corroborated with another detection method (e.g. baiting)**

It was not clear in the paragraph, but we were referring to the detrimental effect of false negative in the context of disease management. In this context, it will be always safer to take measures when none are needed than to not take measures when needed. We tried to clarify that in the text.

Line 316-320 […] However, since these values fall outside the linear part of the standard curve, that 10 copies represent a quantity within the 95% confidence interval, and that, in a disease management context, false negatives are more detrimental than false positives, we decided to use the mean Cq value obtained for 10 copies per reaction as experimentally tested. […]

**Line 311 – include 0 in 0.931. Be careful with , in place of . depending on the journal.**

Answer: Correction made

Line 332-334 […] “The overall regression was statistically significant (R2= 0.937 (multiple) and 0.931 (adjusted), F(3,30)= 148.3, p < 0.001).” […]

**Line 324 – can you please expand on this sentence “Of 488 soil samples, 68 were declared positive for P. abietivora.” How many of the 68 positives were from symptomatic trees and from plantations or natural stands? Were there any negative control soil samples? Were the positive results confirmed with soil baiting (from the previous study in which they were collected)? If so how many were positive with baiting vs this qPCR? Did any of the samples have P. europaea during baiting – i.e. is there any chance that any of the 68 positive trees were positive for P. europaea rather than P. abietivora?**

Answer: we added more information to the text about the repartition of the positive and the overlap with soil baiting. There was no negative control soil sample, as the samples were collected with the objective of determining the causal agent of PRR in plantations. One could argue that the samples from healthy trees from natural forest stands were collected as a form of negative sample, but both qPCR and metabarcoding have shown presence of *P. abietivora* in these samples as well. None of the isolates were identified as *P. europaea*. Current knowledge about *P. europaea* suggest that it is mainly associated with deciduous trees. At the moment, there is no validated report of *P. europaea* in Eastern North America. As mentioned in the discussion, previous reports of P. europaea in Christmas tree plantations should be reevaluated as they are most probably misidentifications.

Line 344-350 […] “Out of the 68, 42 were from PRR symptomatic trees, 17 from healthy trees within the plantations and 9 from trees from natural forest stands near the plantations (Fig 3A). While the low number of strains isolated by baiting preclude any statistical analysis, only a slight overlap was found between the results obtained by baiting and results from the detection of *P. abietivora* DNA in the soil (3 out of 7 samples).” […]

**Line 356, space missing here t(**

Answer: Correction made.

Line 369-370 […] “The average Cq values obtained for soil and root samples were also not significantly different (Welch two sample t-test, t (50.19) = 1.4911, p = 0.14).” […]

**Line 349 – space missing after P.**

Answer: Correction made

Line 372-374 […] “Overall, for 89.2% (282/316) of soil samples, both methods were in agreement on the presence/absence of *P. abietivora*.” […]

**Line 382 – specific to P. abietivora and P. europaea? Semi-specific**

Answer: We added semi-specific in the text.

Line 411-412 […] we developed a sensitive and semi-specific qPCR assay to detect *P. abietivora* in environmental samples (diseased plant tissues and soil).” […]

**Line 397 – italicise Phytophthora**

Answer: Correction made.

Line 424-427 […] “Previous studies have shown that the detection of Phytophthora spp. DNA from soil is feasible even at those low densities [43,44], while other found it harder to effectively detect Phytophthora pathogens due to DNA extraction limitations [45].” […]

**Line 468 – culturing would also enable confirmation of species identification i.e. P. abietivora or P. europaea right?**

Answer: This is correct. But this information is already mentioned earlier in the text.

Line 452-455 […] When the presence of other species from this complex cannot be excluded, this assay should always be coupled with confirmation methods such as strain baiting or strain isolation from diseased tissues, followed by Sanger sequencing of a discriminating locus to confirm the presence of only *P. abietivora*.[…]

General comments:

**Figure 2 D, forest not Forest for title**

Answer: Figure 2D has been updated with *forest*

**Figure 3 A, y axis title more details, Proportion what**

Answer: Figure 3A y-axis has been updated with more details: “Proportion of samples”

**Figure 3 B y axis capital letter missing in Probability**

Answer: Correction has been made.

Reviewer #2:

**The study presents the development of a sensitive and specific qPCR assay targeting Phytopthora abietivora and validating its efficiency on root and in soil samples. The qPCR assay will prove useful in early detection of the pathogen in Christmas tree plantations and of crucial information of the distribution of this emerging species in nature and of a better understanding of its ecology.**

**The ms is well written and each step of the qPCR assay clearly described. The results are presented with adequate statistical methods and described in sufficient detail.**

Answer: We thank the reviewer for their input and their evaluation of our work. We tried to provide a clear and complete answer to each comment raised by the reviewer.

**General comments:**

**The samples used are described as eDNA, however, environmental DNA or eDNA describes the genetic material present in environmental samples such as sediment, water, and air, including whole cells, extracellular DNA and potentially whole organisms. It will be more plain if you explain soil and root samples, eg in the abstract and elsewhere.**

Answer: We agree with the reviewer that we used a rather loose definition of eDNA in our manuscript. We changed the mentions of eDNA with more precise terms when needed.

Line 29-31 […] “This study focused on developing a sensitive and specific qPCR assay targeting *P. abietivora* and validating its efficiency on DNA extracted from soil and roots.” […]

Line 33-34 […] “The assay was tested on DNA obtained from healthy-looking and PRR symptomatic Fraser and balsam firs.” […]

Line 37-38 […] “Out of 488 DNA samples from soil, 68 were positive for *P. abietivora* from which 42 (61.7%) were from PRR symptomatic trees.” […]

Line 401-402 (figure caption) - Fig 4. DNA from *P. abietivora* is found in soil and root fragments samples in comparable quantities

Line 424-427 […] “Previous studies have shown that the detection of Phytophthora spp. DNA from soil is feasible even at those low densities [43,44], while other found it harder to effectively detect Phytophthora pathogens due to DNA extraction limitations [45].” […]

Line 488-489 […] “Large differences in sensitivity between baiting and molecular detection of Phytophthora species in soil samples are not uncommon.” […]

Line 488-492 […] “While more sensitive, using DNA based molecular detection only indicates the presence of the pathogen without any information about its viability status. A positive detection with molecular methods does not always equate to living cells in the sample as it was shown that DNA can persist through time in the environment following the death of organisms [58,59]” […]

**Introduction:
The introduction is very consise and I really miss information about the economic importance of Ornamental plants, and of the estimated loss due to PRR. There is an informative introduction in your article published earlier this year (Survey of Phytopthora Diversity Reveals P. abietivora as a Potential Phytopthora Root Rot Pathogen in Québec Christmas Tree Plantations). Each article must be read independently, so I will appreciate if you can add more information both in the introduction and in the discussion.**

Answer: We acknowledge that our introduction may have been thin on the problematic of Phytophthora in productions. We added some sentences to better inform the reader of the extent of the damage Phytophthora can incur to Christmas tree productions.

Line 47-53 […] “For instance, one of the industries faced with Phytophthora root rot (PRR) is the cultivation of Christmas trees as ornamental plants, where PRR is one of the most prevalent and widely distributed diseases of true firs (genus *Abies*), killing about 10% of the planted Christmas trees in the United States of America [7]. Alone, PRR is responsible for millions of U.S. dollars in yearly losses for this industry, since infected trees are impossible to sell and die within months [8–10].” […]

Line 507-509 […] “In conclusion, this qPCR assay will prove useful in the early detection and biomonitoring of P. abietivora in Christmas trees plantations, hence saving this industry from several millions of dollars in losses.” […]

**Page 4**

**Line 17. Unclear sentence.” In environmental or diseased tissues”**

Answer: There were indeed words missing in this version of the manuscript. We added the missing terms to the sentence.

Line 80-83 […] “Therefore, developing a molecular assay that detects *P. abietivora* in environmental samples or diseased tree samples is of utmost importance for both biomonitoring and diagnostic applications.” […]

**Page 6**

**Line 7 in S1 Table. Please correct.**

Answer: Primers and probe sequences are indeed listed in Table 1, not Table S1. However, Table 1 was misplaced further down in the manuscript. It is now right after the first paragraph where it is mentioned first.

**Page 9**

**Line 6. Please explain what you refer to using environmental samples**

Answer: We were referring to soil sample, we changed the wording to clarify this.

Line 182-183 […] “To estimate the limit for *P. abietivora* quantification in soil samples, oospore suspensions of different concentrations were prepared.” […]

**Page 10**

**Line 5. Without soil? Unclear**

Answer: We clarified the end of the previous paragraph and the beginning where the unclear statement was:

Line 202-204 […] “For each oospore quantity, total DNA extractions were performed on oospores alone or mixed with soil in four replicates.” […]

Line 205-207 […] “For DNA extraction of oospores alone, the oospores were resuspended in 180 µL of ATL buffer from the QIAamp DNA mini kit (Qiagen, Valencia, CA, USA) and DNA was extracted according to the manufacturer’s specifications.” […]

**Line 7. Please describe the origins of the garden soil.**

Answer: The soil was from a lot located in the community garden of our research center. We added a sentence about the origin of the soil in the manuscript. We also performed an extraction on soil alone experiment (without adding oospores) to make sure that *P. abietivora* was not already present in the soil.

Line 210-215 […] “The soil was taken from a lot of the community garden at the Laurentian Forestry Center (46.779, -71.282). To make sure this soil was not previously contaminated with P. abietivora, six independent samples were used for DNA extractions with the Powersoil Pro protocol. On each of the DNA samples obtained, the detection assay was performed in three technical replicates.” […]

**Line 17. A brief recapitulation of the sampling procedure of soil cores will improve the understanding**

Answer: We added a short recapitulation of the sampling to this paragraph.

Line 230-233 […] “In short, at each site, three soil subsamples 120° apart were taken from the crown limit at a 17-cm depth with a soil auger. The organic horizon was removed from the three soil cores and each of them was cut in half lengthwise, one half of cores each core was discarded. The remaining halves were pooled and formed the final sample. [19]” […]

**Line 21. The plantations sampled were of different age?**

Answer: The trees from which soil was sampled were aged between 4 and 12 years. However, this information was only an estimation and no information about the age of each individual tree was recorded. We sampled different plantations between 2019 and 2021. Originally the idea was to sample different locations over 2 years, but covid pandemic made it so the 2020 sampling was split between 2020 and 2021.

**PAGE 11.**

**Line 4. Please add the information on storing the samples prior to analysis.**

Answer: We added the following sentence at the end of the paragraph.

Line 237-239 […] “Prior to DNA extractions, root samples were kept at −20°C between 3 months (2020-2021 samples) up to 2 years (2019 samples) and soil samples were kept at −20°C for up to 2 months.” […]

**Discussion:
Did you find any differences between different ages of plantations?**

Answer: It is an interesting question. However, and as was mentioned earlier, the age of the individual sampled trees was unfortunately not recorded in this study. Moreover, some plantations were even-aged, while others were uneven-aged with restocking between stumps. Therefore, analyse comparing different ages of plantations was not possible.
