# Supplementary material for: A qPCR assay for the detection of Phytophthora abietivora, an emerging pathogen on fir species cultivated as Christmas trees
Source: PLoS One. 2025 Apr 2;20(4):e0320680. doi: 10.1371/journal.pone.0320680 (PMC11964239; doi:10.1371/journal.pone.0320680)
Supplement: Attachment — Submitted filename: PONE-D-24_40058.docx [file pone.0320680.s007.docx]

**A qPRC assay for the detection of Phytopthora abietivora, an emerging pathogen on fir species cultivated as Christmas trees.PONE-D-24-40058**

Comments on the title: Too long

Suggestion: Development of a qPCR assay for the detection of the emerging pathogen P.a on fir species

The study presents the development of a sensitive and specific qPCR assay targeting *Phytopthora abietivora* and validating its efficiency on root and in soil samples. The qPCR assay will prove useful in early detection of the pathogen in Christmas tree plantations and of crucial information o the distribution of this emerging species in nature and of a better understanding of its ecology.

The ms is well written and each step of the qPCR assay clearly described. The results are presented with adequate statistical methods and described in sufficient detail.

General comments:

The samples used are describes as eDNA , however, environmental DNA or eDNA describes the genetic material present in environmental samples such as sediment, water, and air, including whole cells, extracellular DNA and potentially whole organisms. It will be more plain if you explain soil and root samples, eg in the abstract and elsewhere.

Introduction:

The introduction is very consise and I really miss information about the economic importance of Ornamental plants, and of the estimated loss due to PRR. There is an informative introduction in your article published earlier this year (Survey of Phytopthora Diversity Reveals *P. abietivora* as a Potential Phytopthora Root Rot Pathogen in Québec Christmas Tree Plantations). Each article must be read independently, so I will appreciate if you can add more information both in the introduction and in the discussion.

Page 4

Line 17. Unclear sentence.” In environmental or diseased tissues”

Page 6

Line 7 in S1 Table. Please correct.

Page 9

Line 6. Please explain what you refer to using environmental samples

Page 10

Line 5. Without soil? Unclear

Line 7. Please describe the origins of the garden soil.

Line 17. A brief recapitulation of the sampling procedure of soil cores will improve the understanding

Line 21.The plantations sampled were of different age?

PAGE 11.

Line 4. Please add the information on storing the samples prior to analysis.

Discussion:

Did you find any differences between different ages of plantations?
